# Combinatorial Signaling through TLR-2 and CD86 Augments Activation and Differentiation of Resting B Cells

**DOI:** 10.1371/journal.pone.0054392

**Published:** 2013-01-24

**Authors:** Shweta Jain, Sathi Babu Chodisetti, Javed N. Agrewala

**Affiliations:** Immunology Laboratory, Council for Scientific and Industrial Research-Institute of Microbial Technology, Chandigarh, India; University of Miami, United States of America

## Abstract

B cells are an integral component in mounting humoral immune responses and they are also crucial in programming T cell mediated immunity. Usually, B cell activation is initiated by recognition of antigen through B cell receptor (BCR), followed by its processing and presentation to T cells. But very little is known about BCR independent activation of B cells. Now, there is an increasing body of evidence indicating the combinatorial effect of innate and adaptive immune components in modulating the functions of B cells. In this study, we demonstrate the activation of resting B cells (RB) by simultaneous involvement of Toll like Receptor-2 (TLR-2) and costimulatory molecule, CD86. Interestingly, these B cells exhibited significant level of activation and proliferation. Furthermore, this process of activation leads to the differentiation of RB cells, preferably into marginal zone precursors (CD19^+^IgD^hi^IgM^hi^CD21/35^hi^CD23^hi^) in a shorter time window and showed increased secretion of IgG isotype. These RB cells also showed enhanced antigen uptake capacity. These observations were also substantiated by microarray gene expression results, which strengthen the notion that combinatorial signaling through innate and adaptive immune components enhances B cell mediated immune response. Thus, the present study elucidates a novel BCR independent B cell activation mechanism that links TLR-2 and CD86. Hence signaling of TLRs in conjunction with costimulatory molecules will substantially help in bolstering humoral immune response, which can be extrapolated to formulate vaccination strategies for diseases involving B cell-mediated immunity.

## Introduction

It is widely established that two signals are needed for the optimal activation of T cells. Signal-1 involves interaction of antigen specific T cell receptor (TCR) with peptide-major histocompatibility complex (MHC) molecules on the surface of antigen presenting cells (APCs). Signal-2 is also APC driven and engages interaction of costimulatory molecules, mainly CD80 and CD86 with CD28 and CTLA-4 that are expressed on T cells [1]–[3]. The role of costimulatory molecules is well established in the context of T cell activation but not much is known in the case of B cells [4]–[6]. Recently, much evidence has been generated indicating the role of costimulatory molecules in influencing the functions of APCs through bi-directional signaling [7]–[11].

Among the various costimulatory molecules studied, the role of CD86 has been prominently elucidated in affecting the functions of B cells. Direct triggering of B cells through CD86 enhances proliferation, secretion of IgG1 and IgG2a and their survival by augmenting the expression of anti-apoptotic molecules [11]. In addition, cross linking of CD86 on human B cells that are stimulated *in vitro* with CD40 and cytokines enhances secretion of IgE and IgG4 [1]. Similarly, IL-4/CD40 stimulated B cells are synchronously regulated by signaling through CD86 and 2-andregenic receptor. Such B cells exhibit enhanced activation and expression of Oct-2, NF-κB and 3′-H enhancer and have augmented capacity of antibody secretion [9]–[14]. *In vivo* studies have shown that CD86 induces the differentiation of already isotype switched B cells to antibody secreting plasma cells through up regulation of XBP-1 [3]. Further, the role of CD86 has also been demonstrated in germinal center formation and primary humoral response [15]. Moreover, the structural conformation and valence of CD86 confers high affinity for CD28 and therefore it is a preferred ligand over CTLA-4. Interaction of CD86 with CD28 delivers positive signals for T cell and B cell activation [16], [17].

The expression of costimulatory molecules such as CD86 and CD80 on B cells is also augmented by their stimulation through Toll-like receptors (TLRs) [18]–[20]. TLRs are evolutionarily conserved germline encoded molecules that play a key role in regulating innate immune responses. TLRs have gained considerable impetus in influencing the biology of B cells such as their activation, differentiation, antibody secretion and class switch recombination 2,19,21,22. Recent evidence indicates a concurrent effect of molecules of innate immunity such as TLRs and adaptive immunity like BCR, CD38 in the activation and differentiation of B cells. For example, we have recently demonstrated that concerted signaling through CD40 and TLR-2 induces B cell activation and improves their antigen presentation in a BCR-independent manner [7]. Moreover, signaling through TLRs and CD38 or BCR cooperate with each other in inducing activation of B cells [23]–[25]. In addition, sequential signaling through TLR-7 and BCR breaks the tolerance in B cells [26]. Several ligands such as lipoproteins, macrophage-activating lipopeptide-2, neisserial porins, pneumococcal surface adhesin A (PsaA) etc., have been reported to activate B cells through engagement of TLR-2 [27], [28]. The role of TLR-2 on B cells has been implicated in regulating humoral immune responses, in particular, mediating *in vivo* primary humoral immune response [29]. To the best of our knowledge, there is no published evidence indicating the combinatorial effect of CD86 and TLR-2 in regulating the activation of resting B (RB) cells.

In the present study, we have investigated the impact of CD86 costimulation in bolstering the function of TLR-2 elicited RB cells. Our results indicate that this strategy of signaling leads to substantial increase in the activation, differentiation and antigen uptake capacity of RB cells.

## Results

### CD86 costimulation enhances the activation of TLR-2 stimulated RB cells

**Figure 1:**
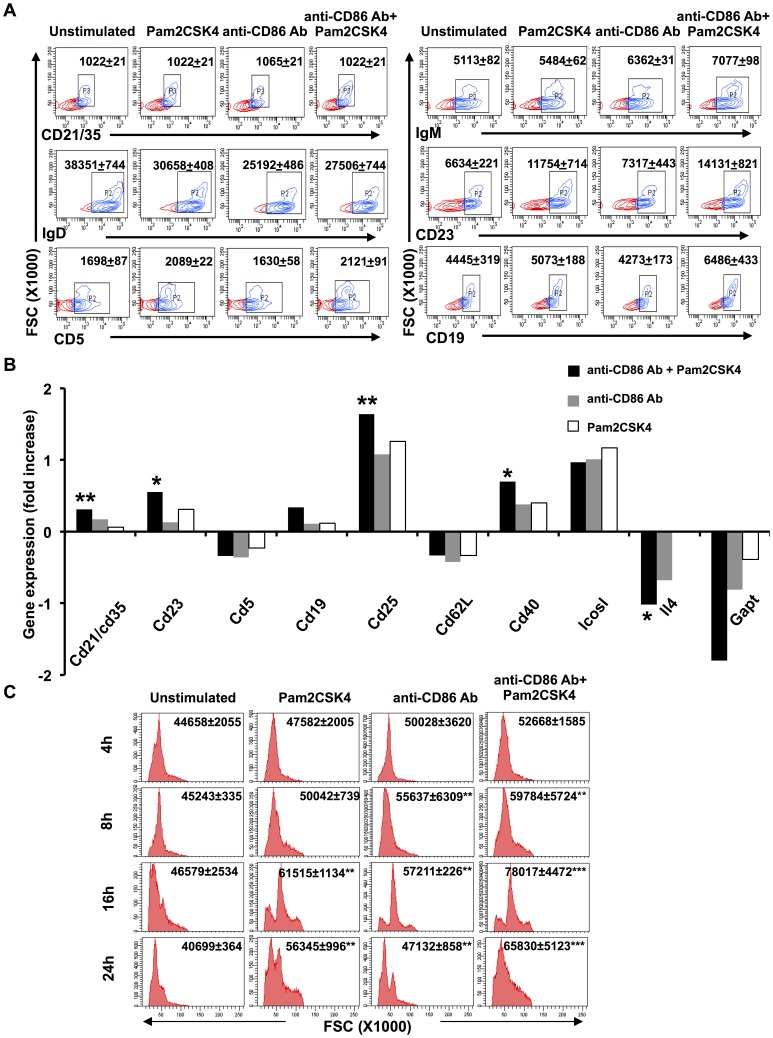
CD86 synergizes with TLR-2 in enhancing the size and activation of RB cells. (A) RB cells from BALB/c mice were stimulated by anti-CD86 Ab and Pam2CSK4 and checked for the expression of CD21/35, IgD, CD5, IgM, CD23 and CD19. Numbers in the flowcytometry plots represent mean fluorescence intensity (MFI) of respective markers; (B) microarray data indicates geometric mean of the fold change in the expression of genes encoding for activation markers. Data are representative of two biological replicates. Statistical analysis was done using One Way ANOVA; (C) change in cell size (FSC) was assessed by flowcytometry. Flowcytometry data (A, C) expressed as mean±SEM are representative of 3–5 independent experiments and statistical analysis was done by Student‘t’ test. ‘*’, ‘**’, ‘***’ indicates p<0.05, p<0.01, p<0.001, respectively.

We first investigated the impact of CD86 and TLR-2 costimulation (TLR2.CD86) on RB cells (Fig. 1A). We observed that TLR2.CD86 stimulation results in an augmented expression of activation markers such as CD21/35, CD23 and IgM. The expression of IgD was downregulated, signifying that these two signals exert conjoining effects on RB cells leading to their activation necessary to counteract antigens. Steady-state equilibrium in RB cells activation is obtained by moderate enhancement in the expression of CD5 and increased expression of CD19 as well as CD21/35. This indicated an improved CD19-mediated signalosome activity that results in reduced threshold of B cell activation. These results correlated well with the microarray gene expression data showing enhanced expression of genes coding for B cell activation markers such as cd21/cd35, cd23, cd19, cd25 and cd40 (Fig. 1B). Moreover, upregulated expression of Cd25 and Cd40 genes indicate mature and activated phenotype. The expression of Gapt gene was found to be reduced on TLR2.CD86 costimulation. Gapt codes for GRB-2 protein, which is a negative regulator of B cell proliferation and survival. An enhancement in the size, proliferation and blast formation was also observed in RB cells upon TLR2.CD86 stimulation (Fig. 1C, Fig. S1, Fig. S2). Kinetics of forward scatter (FSC modulation) revealed that dual signals start affecting the size as early as 4 h and peaked at 16 h (FSC: 46579±2534 *vs.*78017±4472). Taken together, these findings suggested that CD86.TLR2 signals drive B cell maturation and activation.

### Simultaneous signaling through CD86 and TLR-2 enhances the survival of RB cells

**Figure 2:**
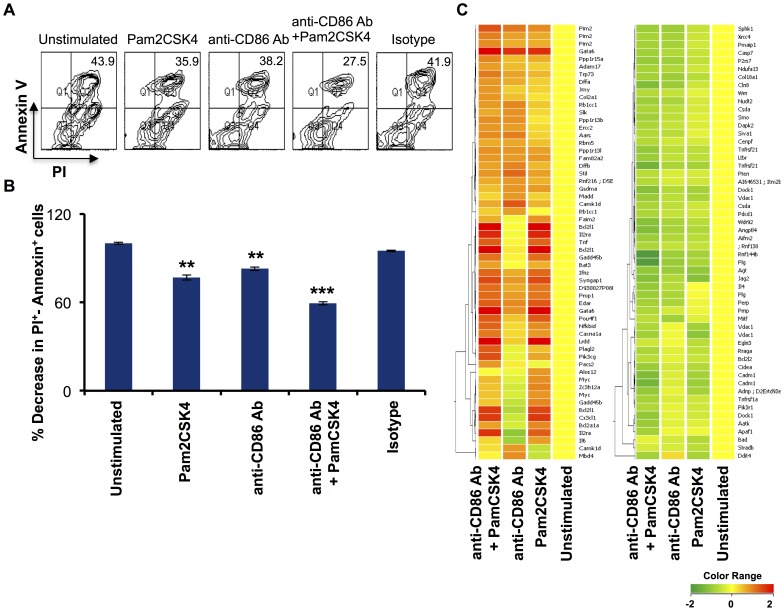
Concomitant signaling of CD86 and TLR-2 reduces the percentage of RB cells undergoing apoptosis. RB cells isolated from BALB/c splenocytes were stimulated by anti-CD86 Ab and Pam2CSK4 for 72 h and apoptosis was checked by PI-Annexin V staining. (A) Flowcytometry density plots show the representative PI-Annexin double positive cell population; (B) bar diagrams indicate percentage change in PI-Annexin double positive population with respect to controls (PI-Annexin double positive cells of unstimulated cells taken as 100%). Flowcytometry data expressed as mean±SEM were representative of 3 independent experiments. Statistical analysis was done by Student‘t’ test and ‘**’, ‘***’ indicate p<0.01 and p<0.001 respectively; (C) dendrograms represent modulation in the expression of genes encoding for molecules involved in apoptosis. Left panel indicates upregulated genes, whereas right panel signify downregulated genes. Scale bar: −2 to +2.

A decreased expression of Gapt gene prompted us to investigate the role of TLR2.CD86 signaling in the survival of RB cells. We stimulated RB cells for 48 hours through both the signals and found that the percentage of cells undergoing apoptosis was drastically reduced when RB cells were stimulated through both CD86 and TLR-2 simultaneously (Fig. 2A, 2B). This further indicated that TLR2. CD86 signaling promotes the survival of RB cells [7], [11]. This observation was supported by microarray data, where we observed that RB cells triggered through TLR2.CD86 had an enhanced expression of genes encoding anti-apoptotic molecules (Bcl21a, Pim2, Lrdd), transcription factors (Gata6), cytokines and their receptors (Il2ra and Ifn), and B cell activation factors (Nfkbid). TLR2.CD86 also led to reduced expression of genes encoding pro-apoptotic factors (Pten, capase-7, capase-3) and apoptosis related genes (Pmaip1,P2rx7, Rnf family of proteins, Dock1, Apaf1, Ddit) (Fig. 2C). Thus, these results suggested that signaling through TLR2.CD86 enhances the survival of RB cells.

### TLR2.CD86 signaling drives RB cell differentiation to marginal zone precursors

**Figure 3:**
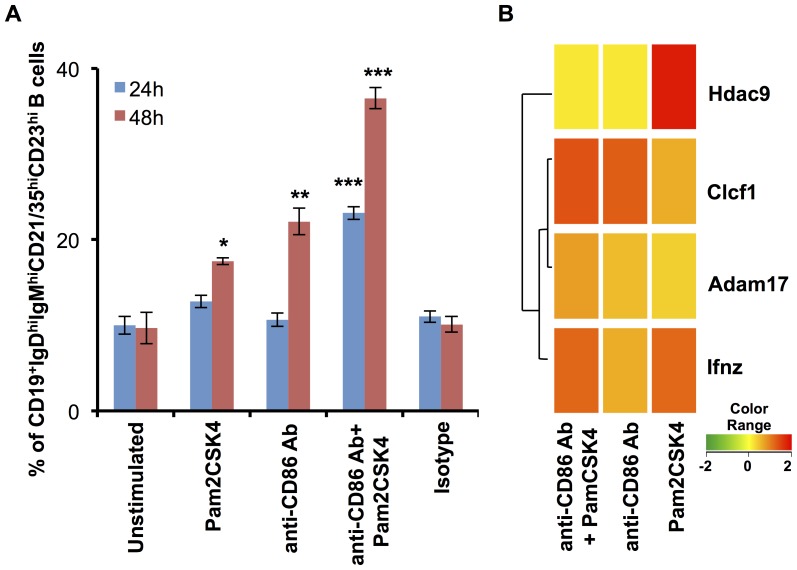
Simultaneous signaling through CD86 and TLR-2 preferentially differentiates RB cells into marginal zone cells. Signaling was delivered in RB cells isolated from BALB/c with anti-CD86 Ab and Pam2CSK4. (A) Bar diagrams show marginal zone precursors (CD19^+^, IgD^hi^IgM^hi^CD21/35^hi^CD23^hi^) in differentially stimulated RB cells at 24 h and 48 h. Values indicate the mean±SEM of percent populations of IgD^hi^IgM^hi^CD21/35^hi^CD23^hi^ expressing cells. Data are representative of three independent experiments. Statistical analysis was done by Student‘t’ test. ‘*’, ‘**’, ‘***’ indicates p<0.05, p<0.01, p<0.001, respectively. (B) Dendrograms depict upregulation in genes involved in differentiation of RB cells with respect to unstimulated controls in different color codes (yellow: no change, red: upregulation, green: downregulation). The values indicate geometric mean of fold change of two of the biological replicates.

As stated previously, TLR2.CD86 signaling leads to an enhanced expression of Gata6 gene. This indicated that these signals have impact on terminal B cell differentiation and proliferation. Hence we examined the outcome of TLR2.CD86 stimulation on differentiation of RB cells. We noted that TLR2.CD86 stimulated RB cells preferentially differentiate into marginal zone precursors (MZP; CD19^+^IgD^hi^IgM^hi^CD21/35^hi^CD23^hi^), which may later form marginal zone cells. We observed a substantial enhancement in the percentage of MZP cells (10% to 36% at 48 h) when RB cells were stimulated through TLR2.CD86 (Fig. 3A, Fig. S3). This enhancement was observed from an early time (24 h), indicating that TLR2.CD86 signals reduce the differentiation period/window of RB cells. In support of this finding, we observed upregulated gene expression of Clcf1, a neurotrophic factor that activates JAK/STAT pathway, which is involved in B cell activation and differentiation (Fig. 3B). The significance of this observation lies in the fact that MZ B cells are non-circulating mature cells that can be rapidly recruited in an early adaptive immune response in a T cell dependent manner. These are innate B cells with a lower activation threshold and a higher propensity of differentiation into plasma cells, thereby further accelerating primary antibody response [30]. TLR2.CD86 signaling of RB cells amplifies this population, thereby further aiding in their transformation into plasma cells and antibody secretion. Hence, TLR-2 and CD86 not only bridge innate and adaptive immunity but also amplify the magnitude of immune responses, which may help in boosting the B cell-mediated immune response.

### Stimulation through TLR2.CD86 boosts antibody secretion and class switch recombination of RB cells

**Figure 4:**
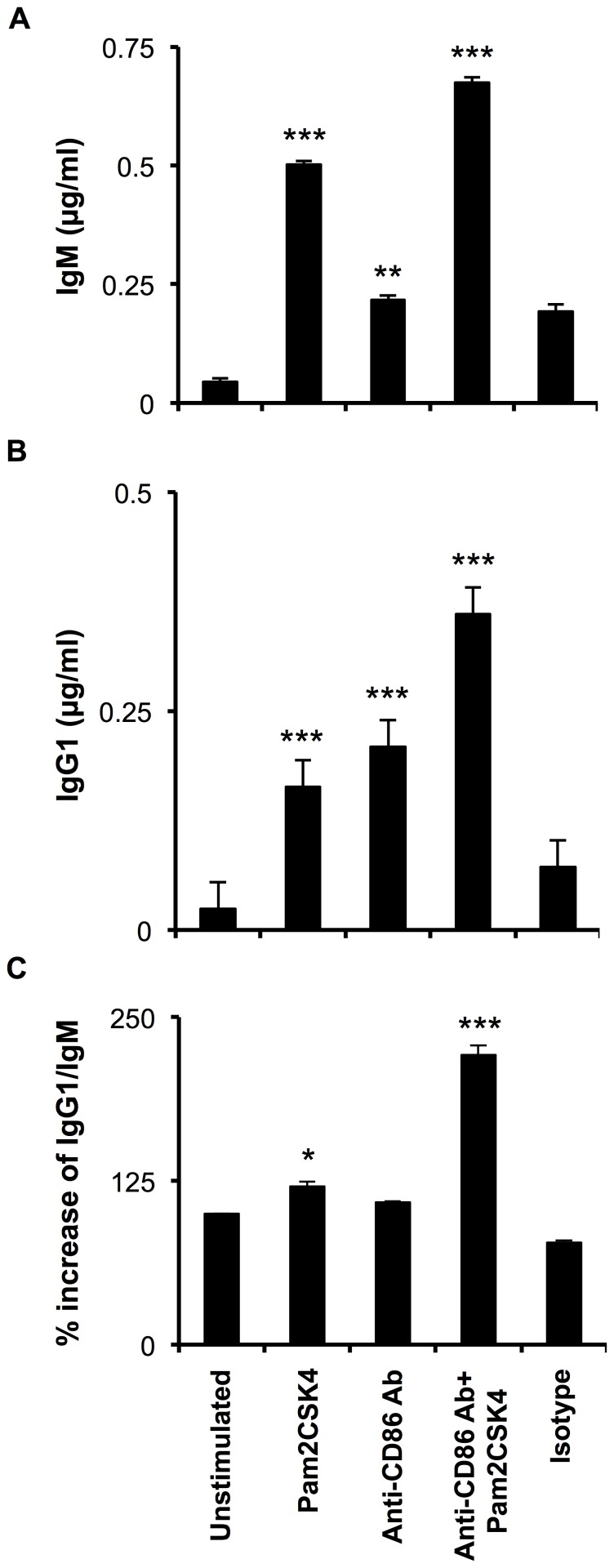
CD86 show synergism with TLR-2 in augmenting the secretion of IgM and IgG1 and class switch recombination. (A, B) Supernatants from 5–6 days old cultures of RB cells isolated from BALB/c mice and stimulated with anti-CD86 Ab and Pam2CSK4 were harvested and assessed for the production of IgM and IgG1 by sandwich ELISA. Data expressed as µg/ml represent mean ± SEM of the triplicate wells were calculated with respect to standards. (C) Ratio of surface expression of IgG1 and IgM on RB cells stimulated with anti-CD86 or isotype antibody in the presence or absence of Pam2CSK4 for 16 h. Data are representative of 3–4 experiments. (A, B, C) Statistical analysis is done by non-parametric Mann-Whitney two tailed test and repeated measure ANOVA with post Student-Newman-Keuls multiple comparisons test by Graph Pad InStat 3 software. ‘p’ values are denoted with respect to unstimulated controls. ‘*’, ‘**’, ‘***’ indicates p<0.05, p<0.01, p<0.001, respectively.

We observed that TLR2.CD86 signaling preferentially transforms RB cells into MZP which, in turn, promotes differentiation of plasma cells. Hence it was imperative to assess the role of TLR2.CD86 signaling in immunoglobulin secretion and class switch recombination (CSR). TLR2.CD86 stimulated RB cells secreted elevated levels of IgM and IgG1 (Fig. 4A, B). The ratio of CSR in such cells was calculated as the ratio of surface expression of IgG1 to IgM. TLR2.CD86 stimulated RB cells showed enhanced CSR ratio. This indicated that TLR2.CD86 signals are effective in CSR (Fig. 4C). This response was significantly better than that observed in the control cells stimulated individually through TLR-2 or CD86. Thus, it may be inferred that RB cells acquire improved levels of activation and maturation and secrete elevated levels of immunoglobulin isotypes when activated through TLR2.CD86.

### TLR-2.CD86 stimulated RB cells have enhanced antigen uptake abilities

**Figure 5:**
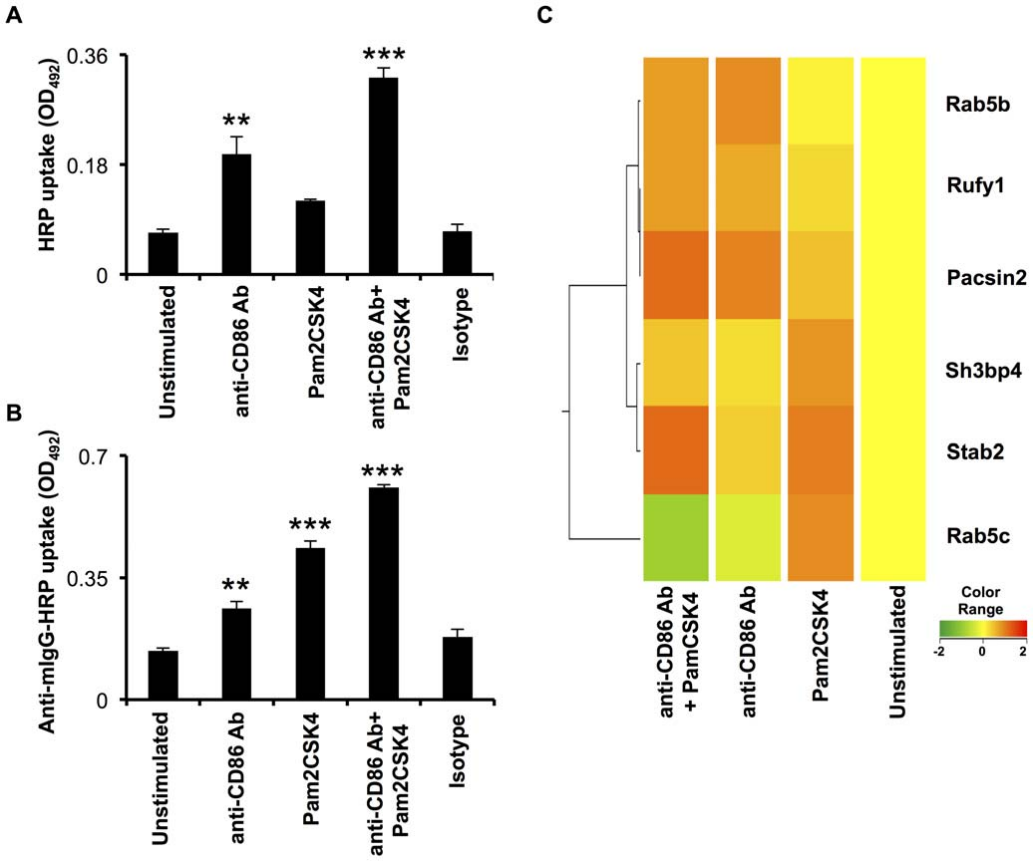
Triggering through CD86 and TLR-2 enhances pinocytosis and receptor mediated endocytosis abilities of RB cells. (A, B) RB cells of BALB/c mice were stimulated with anti-CD86 Ab and Pam2CSK4 and pulsed with (A) soluble HRP and (B) anti-mouse IgG-HRP. The antigen was chased at 37°C for 30 minutes and internalization of antigen was determined colorimetrically. Values shown in the graphs were normalized against experimental blanks and control cells (maintained at 4°C and lysed). The data represents mean ± SEM of one of the 2–3 individual experiments. ‘*’, ‘**’, ‘***’ indicates p<0.05, p<0.01, p<0.001, respectively. (C) Dendrograms represent modulation in the expression of genes encoding for molecules involved in endocytosis. Scale bar: −2 to +2.

We next monitored whether TLR2.CD86 stimulated RB cells gain improved antigen uptake through pinocytosis and receptor mediated endocytosis. We observed that TLR2.CD86 stimulated RB cells acquired enhanced pinocytotic ability when pulsed with horse radish peroxidase (HRP), as a soluble antigen (Fig. 5A). Further, TLR2.CD86 stimulated RB cells also showed marked increase in receptor-mediated endocytosis of anti-mouse IgG-HRP antibody. This response was significantly better than the controls where signal was delivered through TLR-2 or CD86 individually (Fig. 5B). Enhanced antigen uptake by TLR2.CD86 stimulated RB cells may also be due to augmented expression of Rab genes, which are responsible for actively promoting endocytosis. In addition, we also observed upregulation in genes such as Pascin2 and Stab2, which are involved in tubulin polymerization, rearrangement of cytoskeleton and endocytosis of low density antigens (Fig. 5C). Hence, we concluded that TLR2.CD86 signaling not only improves the activation and maturation of RB cells, but also enhances their antigen uptake ability.

## Discussion

In the current state of events, expanding evidence indicates synchronous functioning of molecules responsible for modulating innate and adaptive immune response. Studies have shown that various components of immune system like NK cells, dendritic cells, TLRs, complement system, glycans and defensins play a fundamental role in bridging the innate and adaptive immune response.

Recently, we have demonstrated that CD40 and TLR-2 combine to bring together the adaptive and innate arms of the immune system [7]. Yet, the nexus of innate and adaptive immunity still curtails many facets. Ongoing efforts are directed to explore new molecules or to define novel roles of already established molecules that can further cement the gap between these two limbs of immune system.

In a continual pursuit, we report here a unique role of combinatorial signaling through TLR-2 and CD86 in modulating the functions of RB cells. Our major findings suggest that TLR2.CD86 stimulated RB cells exhibit upregulated expression of activation markers and enhanced survival. Such RB cells differentiate into marginal zone precursors and express enhanced class switch recombination. Moreover, they also have improved ability of antigen uptake. Our studies involved highly enriched RB cells with no contaminating T cell populations. This ruled out any possibility of T cell mediated B cell activation. Moreover, because we used Pam2CSK4 ligand and anti-CD86 antibody for signaling to ensure that the observed experimental outcomes were solely due to signaling through TLR-2 and CD86. These findings suggest a distinct possibility for the existence of BCR-independent pathway for activation of B cells by costimulatory molecules and TLRs. Our findings also demonstrate the cooperative role of the molecules of innate and adaptive immunity in activating RB cells.

Signaling through CD86 has positive effects on B cell functions in terms of their proliferation, immunoglobulin isotype secretion and survival [3], [7], [11], [14]. The expression of CD86 is enhanced on B cells following their stimulation with TLRs [18]. Moreover, TLRs also play an instrumental role in boosting the functions of B cells [2]. There is an increasing body of evidence, which indicates that simultaneous signaling through TLRs and molecules of innate immunity such as CD38 induce strong proliferative and activation signals in B cells [31]. In line with these observations and our own findings, which infer that CD40 also synergizes with TLR-2 in augmenting B cell activity [7], we further sought to ascertain the effect of concomitant signaling through CD86 and TLR-2 on RB cells. Intriguingly, we found that such stimulation could bolster the activation status of B cells in terms of enlargement of size and expression of activation markers such as CD21/35, CD23 and CD19. The activation process does not happen in a slap dash or haphazard manner because there is slight upregulation in the expression of negative regulators of B cells such as CD5. Thus, the entire plethora of molecules such as CD23, CD21, IgM, IgD, CD5, etc., aid in maintaining the immune homeostasis, which indicates that these signals evoke events that are responsible for regulated activation of RB cells. As such, RB cells have little costimulatory activity as compared to mature B cells due to lesser expression of CD86. Yet, signaling through CD86 can result in an enhanced activated phenotype. When this signaling is coupled with another positive modulator, such as TLR-2, both function conjunctively to modulate the activity of B cell.

Concurrent signaling through CD86 and TLR-2 also led to B cell differentiation into marginal zone precursors (MZP). These MZP later differentiate into marginal zone cells that again act to connect innate and adaptive immunity. The lower threshold of activation of marginal zone cells and their easy propensity to differentiate into plasma cells makes them suitable for effector function, in a lesser time span. It also leads to enhanced maturation of RB cells, which undergo more class switch recombination and secrete elevated levels of immunoglobulin isotypes. This convinced us to put forward a novel concept that combined triggering of TLR-2 and CD86 can elicit enhanced secretion of antibodies, thereby boosting humoral immunity.

The inherent ability of B cells to act as antigen presenting cells (APC) is usually remote. They cannot uptake, process and present antigen as efficiently as dendritic cells. Signaling through CD86 and TLR-2 augmented the ability of B cells to acquire antigen through both fluid-phase pinocytosis and receptor-mediated endocytosis. These observations gave clear implications that CD86 and TLR-2 can act in cohort to not only achieve optimum activation functions of B cells, but also to improve their role as APC. This study, therefore, demonstrates that CD86 in association with TLR-2 can trigger B cells in a BCR independent fashion. We propose a novel role of simultaneous signaling through CD86 and TLR-2 in RB cells to significantly improve their functions as humoral mediators of immune response as well as antigen presenting cells. Our findings open avenues to investigate the molecular mechanisms involved in the dynamics of such concurrent signaling that may decipher these results more comprehensively. Finally, triggering through TLR2 and CD86 can be employed in bolstering the efficacy of B cell-mediated vaccines.

## Conclusions

The present study indicates that there exists a flexible continuum between molecules of innate and adaptive immunity and both of them work in concert to maintain a homeostatic harmony. Simultaneous signaling through CD86 and TLR-2 unravels a unique BCR independent mechanism of activating resting B cells. Cells stimulated in this manner have an enhanced longevity, activation profile, differentiation status and ability to uptake antigen. The data are in agreement with the gene expression profiles and insinuates to further decipher the molecular mechanism behind this concurrence. An insight into the detailed mechanism may pave way to design vaccines based on this signaling strategy.

## Methods

### Ethics statement

Male or female BALB/c, C57BL/6 and C3He mice of 6–8 weeks were procured from National Institute of Immunology, New Delhi and National Institute of Pharmacological Education and Research, Mohali and National Institute of Nutrition, Hyderabad, India. All animal studies were approved by the Institutional Animal Ethics Committee of Institute of Microbial Technology (Ref. No 55/1999/CPCSEA), India.

### Reagents

B cell cocktails, biotin-conjugated anti-mouse CD43 (S7), CD45R (B220, RA3-6B2), CD40 (3/23), isotype control (IgG2aκ,R35-95), CD23 (B3B4); PE-labeled anti-mouse IgD (11-26c.2a), CD86 (GL1); PE-Cy5 conjugated CD5 (53-7.3), PE-Cy7 coupled IgM (R6-60.2); APC or PE-Cy5 conjugated streptavidin; APC-Cy7 linked CD19 (1D3) and Pacific blue tagged CD45R (B220, RA3-6B2) and all ELISA reagents were purchased from BD Biosciences (San Diego, CA). TLR-2 ligand (Pam2CSK4) was obtained from Invivogen (San Diego, CA). RPMI-1640 and FCS were from Gibco (Grand Island, NY). All other standard reagents were procured from Sigma (St. Louis, MO), unless otherwise mentioned.

### B cell isolation and stimulation

RB cells were purified from mice (BALB/c or C57BL/6) splenocytes using CD43 negative B cell isolation method, as described previously (7). Briefly, after preparing single cell suspension, splenocytes were treated with B cell enrichment cocktail supplemented with biotinylated anti-CD43 Abs. Later, same volume of streptavidin-magnetic beads was added and RB cells were negatively selected on BD IMagnet. The purity of RB cells obtained was >98%, as ascertained by flow cytometry (CD45R^+^CD19^+^CD43^−^CD4^−^CD8^−^). Enriched RB cells were incubated with biotin conjugated anti-CD86 Abs and isotype matched control (IgG2a,κ) (0.5 µg/10^6^ cells) for 30 minutes on ice followed by cross-linking with equivalent concentration of streptavidin (0.5 µg/10^6^ cells), under similar conditions. The cells were cultured in RPMI-1640 (10% FCS) with specified concentration of Pam2CSK4 (50 ng/ml). Appropriate controls such as cells alone, cells stimulated with anti-CD86 Abs alone or isotype matched Abs alone were also kept in all experiments.

### Flow cytometry

Cells were stained with fluorochrome labeled anti-mouse CD21/35, CD5, IgD, IgM, CD19 Abs and their respective isotype matched controls, as described previously (7). Finally, cells were washed and fixed in 1% paraformaldehyde. The flowcytometry data was acquired on either on BD FACS Calibur or on BD FACS Aria II using Cell Quest Pro (FACS Calibur) or FACS Diva software (FACS Aria II). Data was analyzed by FACS Diva software, as described previously (7). Flowcytometry data is either represented as percentage population or as mean fluorescence intensity (MFI) normalized with suitable isotype matched controls. FSC in flowcytometry data represents cell size, depicted on a linear scale.

### Isotype ELISA

Supernatants were collected from 5–6 day old cultures of RB cells (2×10^5^ cells per well) after stimulation with anti-CD86 Ab (0.5 µg/10^6^ cells) with or without Pam2CSK4 (50 ng/ml) and assayed for IgM and IgG antibodies, as described previously (7). In brief, ELISA plates were coated with purified rat anti-mouse IgM or IgG1 Abs at 4°C overnight. The plates were then washed 3× with buffer (PBS+0.05% Tween-20) and blocked with PBS+1% BSA for 2 h at room temperature and washed again 3×. Culture supernatants and serial dilutions of the standards (purified mouse IgM or IgG1; log_2_ dilutions) were added in these plates and incubated at 4°C over night. Plates were again washed and bound IgM and IgG1 were captured by secondary biotinylated anti-mouse IgM and IgG1 Abs, respectively, followed by their detection with avidin-HRP/OPD-H_2_O_2_ for colorimetric estimation. The concentrations of isotype antibodies secreted were calculated using optical densities (ODs) of standard IgM and IgG1 as reference curves and results were expressed as µg/ml.

### Antigen uptake assay

Antigen uptake assays were performed according to the methods described previously (7). In brief, signaling was delivered in RB cells (2×10^5^/well) using anti-CD86 Abs (0.5 µg/10^6^ cells) in the presence or absence of Pam2CSK4 (50 ng/ml) for 16 h at 37°C. The cells were harvested, washed and incubated with either anti-mouse IgG-HRP Ab (1∶100) or free HRP (200 µg/ml) and incubated on ice for 15 minutes followed by culturing in RPMI-FBS-5% at 37°C. Antigen uptake kinetics was done for 30–60 minutes and was later stopped by adding chilled PBS. Cells were washed thoroughly with ice cold PBS (containing 1% FBS) and subsequently they were treated with pronase. The cells were washed and lysed with a mixture of Tris-HCl (10 mM) and Triton X-100 (0.05%) for 30 minutes on ice, with intermittent vortexing. Intracellular HRP in the cell lysates was estimated colorimetrically (at 492 nm) using OPD-H_2_O_2_ chromogen-substrate method. Cells treated with pronase, cells maintained at 4°C, and unlysed cells were taken as controls. The presence of HRP in the controls was suitably normalized with test samples.

### Propidium Iodide (PI) -Annexin Assay

The assay was performed according to standard procedures, as described previously (7). In brief, RB cells (5×10^4^/well) were stimulated with anti-CD86 Ab (0.5 µg/10^6^ cells) in the presence or absence of Pam2CSK4 (50 ng/ml) for 48 h at 37°C in 200 µl of RPMI-FBS-10%. The cells were harvested, washed and re-suspended in binding buffer [0.01 M HEPES (pH 7.4), 0.14 M NaCl, and 2.5 mM CaCl_2_]. FITC conjugated Annexin V antibody (5 µl/tube) and propidium iodide (0.25 µg/5 µl) were added to the cells. The cells were incubated in dark for 15 minutes at room temperature. Later, binding buffer (400 µl) was added and cells were acquired immediately on BD FACS Calibur flowcytometer using Cell Quest Pro software. Data were analyzed by BD FACS Diva software.

### Microarray analysis

Microarray experiments were done by Genotypic Technology Pvt. Ltd. (Bangalore, India; www.genotypic.co.in). RB cells from BALB/c mice were stimulated with Pam2CSK4 (50 ng/ml) in the presence or absence of anti-CD86 Ab or isotype control (0.5 µg/10^6^ cells). RNA was isolated, quantified and stored frozen until use, as described previously (7). The isolated RNA samples were labeled with Cy-3 using Agilent Quick-Amp labeling kit (p/n5190-0442) according to manufacturer's instructions. 800 ng of Cy-3 labeled cRNA samples were fragmented and hybridized on to a Genotypic designed Custom Whole Genome Mouse 8×60 k (AMADID No: 26986). Fragmentation of labeled cRNA and hybridization were performed using the gene expression hybridization kit of Agilent. Hybridization was done in Agilent's Surehyb Chambers for 16 h at 65°C. The hybridized slides were washed using Agilent gene expression wash buffers and scanned with Agilent microarray scanner (G2505C) at 3 µ resolution. Data extraction from images was done employing Feature extraction software v 10.5.1.1 of Agilent. Further, feature extracted data were analyzed using Gene Spring GX v 11 software of Agilent. Genes were classified based on the functional category and pathways using software Gene Spring GX and Genotypic Biointerpreter-Biological Analysis. Microarray data has been submitted to GEO data base (accession number - GSE28517).

### Statistics

The statistical analysis of the data was done by Student's ‘t’ test, non-parametric Mann-Whitney two tailed and repeated measure ANOVA with post Student-Newman-Keuls multiple comparisons test using Graph Pad InStat 3 software. Statistical analysis of microarray data was done using one way ANOVA.

## Supporting Information

Figure S1 **CD86.TLR-2 stimulation augments the proliferation of RB cells.** Signaling was delivered in RB cells isolated from C57BL/6 mice for 24 h with anti-CD86 Ab (0.5 µg/10^6^cells) and different concentrations of Pam2CSK4. Later, ^3^H-thymidine was added and cells were further incubated for additional 16 h. Cells were harvested and the amount of radioactivity incorporated was measured by liquid scintillation counting. Data are represented as the counts per minute (cpm) and expressed as mean ± SD from triplicate wells. Results are indicative of two independent experiments. ‘*’, ‘**’, ‘***’ indicate p<0.05, p<0.01, p<0.001, respectively.
(TIF)

Figure S2 **Concomitant signaling through CD86 and TLR-2 induces blast formation in resting B cells.** Signaling through CD86 and TLR-2 was delivered in RB cells with anti-CD86 Ab and Pam2CSK4, either separately or in conjunction for indicated durations. Bright field images of the cultures were taken after stipulated durations of stimulation at 40×1.6 magnification using a constant exposure time (11.11 sec). For each combination, 5–6 different fields were imaged. Shown here are the images from representative of three independent experiments.
(TIF)

Figure S3 **Sequential gating of resting B cells to define MZP cell subsets.** (A) CD19^+^ lymphocytes were further gated on the basis of expression of IgD and IgM and defined as follicular cells I and II (FO I, II), marginal zone cells (MZ) and marginal zone precursors (MZP). IgD^hi^IgM^hi^ cells were further differentiated into marginal zone precursors (MZP) on the basis of CD21/35 and CD23 expression; (B) contour diagrams of marginal zone precursors in differentially stimulated B cells at indicated time durations. Values in contour plots indicate the percent populations of IgD^hi^IgM^hi^CD21/35^hi^CD23^hi^ expressing cells. Data are representative of three independent experiments.
(TIF)
