# Correction: Combinatorial Signaling through TLR-2 and CD86 Augments Activation and Differentiation of Resting B Cells

**DOI:** 10.1371/annotation/f0ad0323-84d6-4c21-be15-d07aed1993a1

**Published:** 2014-01-09

**Authors:** Shweta Jain, Sathi Babu Chodisetti, Javed N. Agrewala

An error occurred in Figure 1A, in the first panel of flowcytometry plots depicting change in the expression of CD21/35 versus FSC in resting B cells upon concomitant signaling. The correct values for these plots should read as 1022+21 (unstimulated); 1065+31(Pam2CSK4); 1035+33 (anti-CD86 Ab) and 1277+27(anti-CD86 Ab+Pam2CSK4). 

Please view the correct version of Figure 1 here: http://plosone.org/corrections/pone.0054392.g001.cn.tif

