# Supplementary figures and images for: Combinatorial Signaling through TLR-2 and CD86 Augments Activation and Differentiation of Resting B Cells
Source: PLoS One. 2013 Jan 24;8(1):e54392. doi: 10.1371/journal.pone.0054392 (PMC3554778; doi:10.1371/journal.pone.0054392)

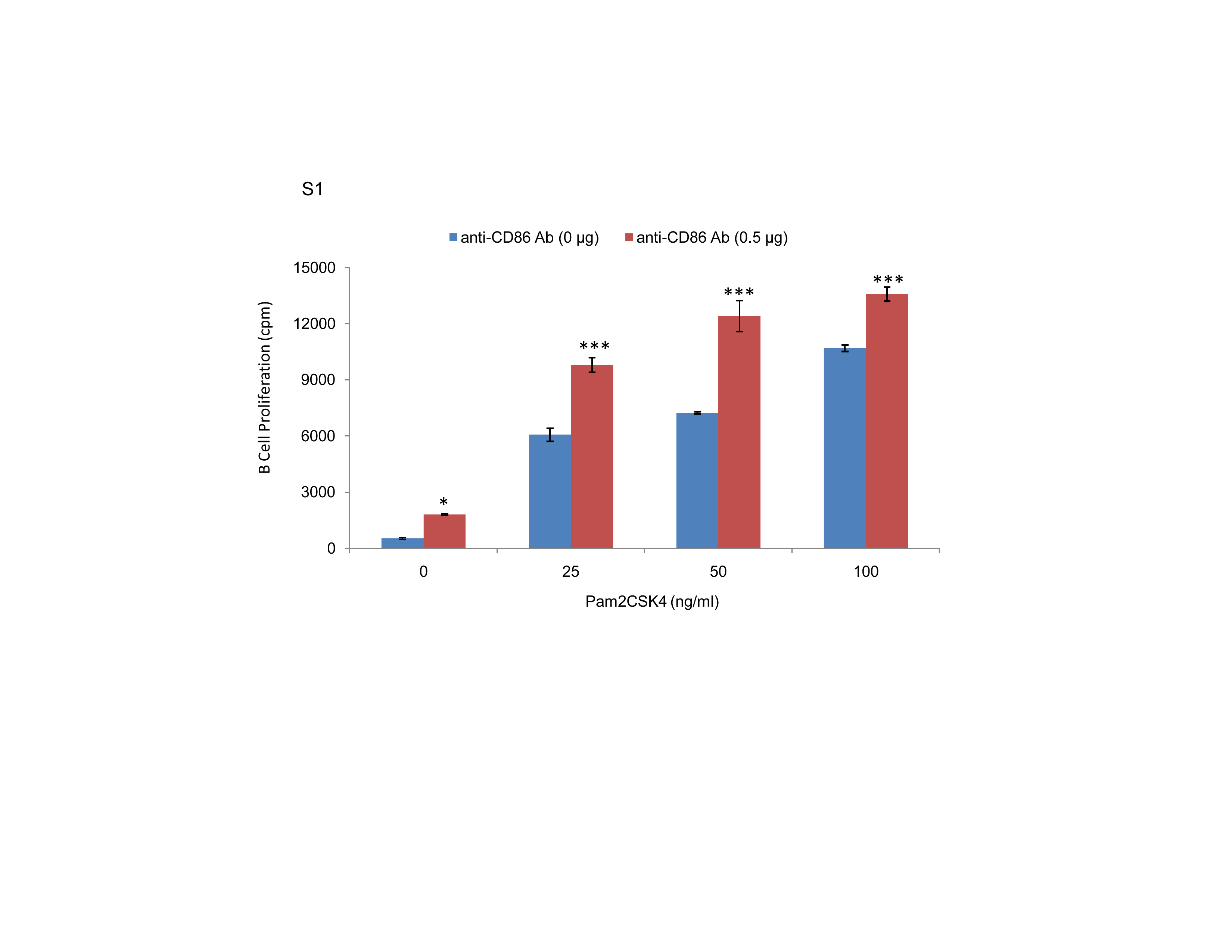

Supplement: Figure S1 — CD86.TLR-2 stimulation augments the proliferation of RB cells. Signaling was delivered in RB cells isolated from C57BL/6 mice for 24 h with anti-CD86 Ab (0.5 µg/106cells) and different concentrations of Pam2CSK4. Later, 3H-thymidine was added and cells were further incubated for additional 16 h. Cells were harvested and the amount of radioactivity incorporated was measured by liquid scintillation counting. Data are represented as the counts per minute (cpm) and expressed as mean ± SD from triplicate wells. Results are indicative of two independent experiments. ‘*’, ‘**’, ‘***’ indicate p<0.05, p<0.01, p<0.001, respectively. (TIF) [file pone.0054392.s001.tif]

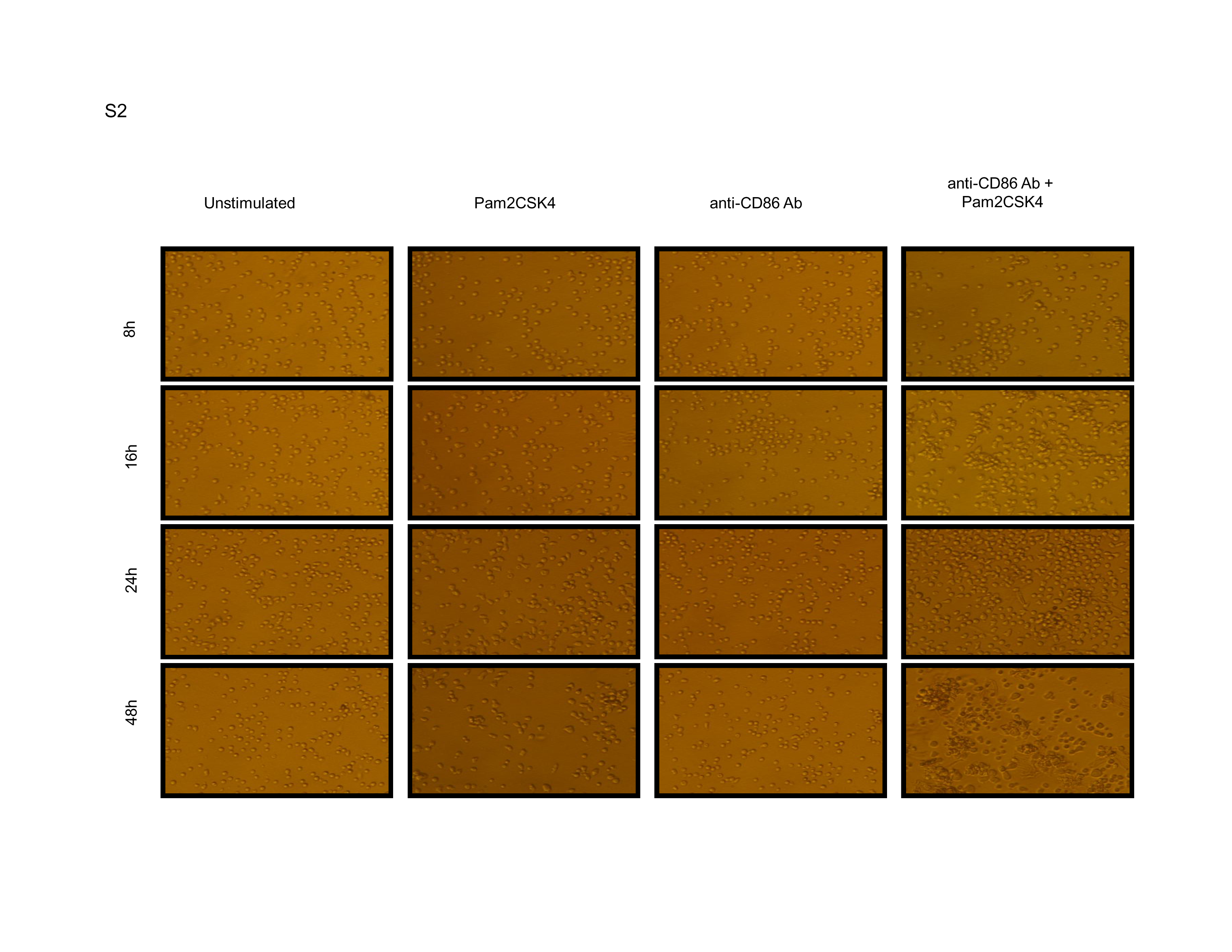

Supplement: Figure S2 — Concomitant signaling through CD86 and TLR-2 induces blast formation in resting B cells. Signaling through CD86 and TLR-2 was delivered in RB cells with anti-CD86 Ab and Pam2CSK4, either separately or in conjunction for indicated durations. Bright field images of the cultures were taken after stipulated durations of stimulation at 40×1.6 magnification using a constant exposure time (11.11 sec). For each combination, 5–6 different fields were imaged. Shown here are the images from representative of three independent experiments. (TIF) [file pone.0054392.s002.tif]

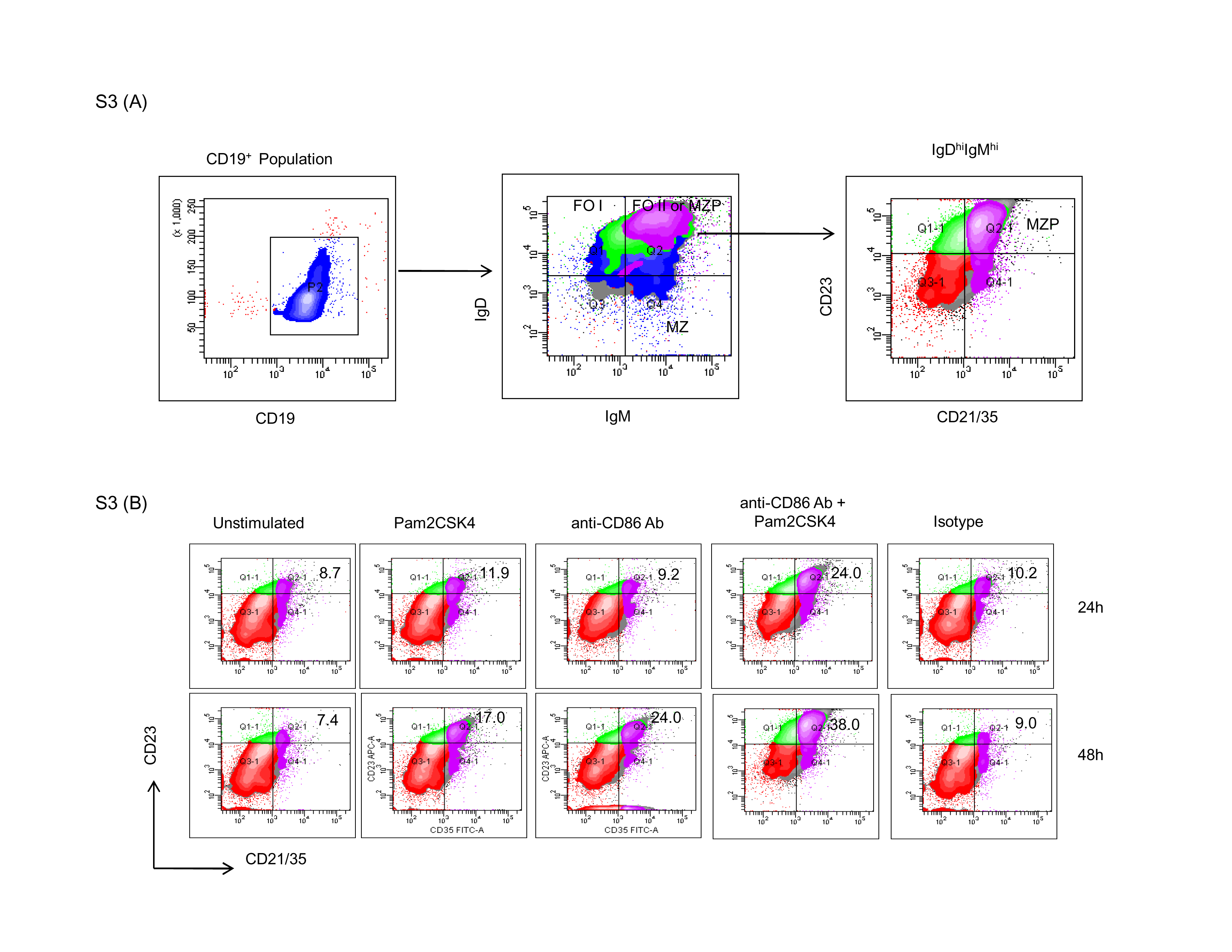

Supplement: Figure S3 — Sequential gating of resting B cells to define MZP cell subsets. (A) CD19+ lymphocytes were further gated on the basis of expression of IgD and IgM and defined as follicular cells I and II (FO I, II), marginal zone cells (MZ) and marginal zone precursors (MZP). IgDhiIgMhi cells were further differentiated into marginal zone precursors (MZP) on the basis of CD21/35 and CD23 expression; (B) contour diagrams of marginal zone precursors in differentially stimulated B cells at indicated time durations. Values in contour plots indicate the percent populations of IgDhiIgMhiCD21/35hiCD23hi expressing cells. Data are representative of three independent experiments. (TIF) [file pone.0054392.s003.tif]
